# How can quality be measured within a physician-led Community Emergency Medical service? A scoping review protocol

**DOI:** 10.1186/s13643-023-02424-w

**Published:** 2024-01-02

**Authors:** Jamie Scott, Libby Thomas, Tony Joy, Paddy McCrossan

**Affiliations:** 1 Physician Response Unit, Barts Health NHS Trust, London, UK; 2 Kings College Hospital NHS Foundation Trust, London, England; 3 Queen’s University Belfast, Belfast, UK

**Keywords:** Quality indicators, Emergency medicine, Pre-hospital, Community Emergency Medicine, Key performance indicators

## Abstract

**Background:**

Quality measurement as part of quality improvement in healthcare is integral for service delivery and development. This is particularly pertinent for health services that deliver care in ways that differ from traditional practice. Community Emergency Medicine (CEM) is a novel and evolving concept of care delivered by services in parts of the UK and Ireland. This scoping review aims to provide a broad overview of how quality may be measured within services delivering CEM.

**Methods and analysis:**

The methodology follows both the Preferred Reporting Items for Systematic Review and Meta-Analysis extension for Scoping Reviews (PRISMA-ScR). It is guided by recognised work of Arksey and O’Malley and the guidelines developed by the Joanna Briggs Institute. Several databases will be searched: MEDLINE, EMbase, EMcare, CINAHL, Scopus, the Cochrane Library and grey literature. Search terms have been developed by representatives within Community Emergency Medicine services. Two reviewers will independently screen eligible studies for final study selection. Results will be collected and analysed in descriptive and tabular form to illustrate the breadth of quality indicators that may be applicable to CEM services. This scoping review protocol has been registered with the Open Science Framework platform (osf.io/e7qxg).

**Discussion:**

This is the first stage of a larger research study aimed at developing national quality indicators for CEM. The purpose of this scoping review is to provide a comprehensive review of quality indicators that could be used within CEM. The results will be mapped using a framework and identify gaps in the literature to help guide future-focused research.

**Supplementary Information:**

The online version contains supplementary material available at 10.1186/s13643-023-02424-w.

## Background

Community Emergency Medicine (CEM) is a novel model of emergency care that has evolved within pre-hospital care [1]. Traditionally, pre-hospital care is emergency medical care that is delivered to patients prior to arrival in hospital following the activation of emergency medical services [2]. Within the United Kingdom (UK) this is predominantly delivered by the National Health Service (NHS) Ambulance Service Trusts.

Pre-hospital care is continuously evolving, novel concepts of care have helped redefine the scope of traditional pre-hospital care [2]. Specialist paramedics and ‘physician-delivered pre-hospital emergency medicine’ are increasingly utilised to target defined cohorts of patients [3, 4]. Their care is tailored to the needs of the patient and considers how the patient may be managed within the systems of hospitals and community care [2–4].

PHEM is an established specialty that delivers advanced medical care and safe hospital transfer for seriously ill and injured patients [5]. CEM has evolved from PHEM after it was recognised that there were emergency needs beyond critical illness that could benefit from specialist physician care. It is the clinical or situational complexity of the patient, rather than the acuity of illness, that defines the cohort of emergency patients targeted by CEM. The complexity of the patient and their care needs are believed to challenge traditional pre-hospital care models, where physician-led intervention results in outcomes that are beneficial for both the patient and the health system [6, 7].

A universally shared CEM model of care has yet to be formally defined. In 2021 Hanks, Ramage and Leech described and evaluated the practices of five services that are delivering CEM across the UK and Ireland [1]. These services deliver a definitive assessment of patients with emergency care needs by clinicians empowered by knowledge, training, equipment and integrated care structures, independent of the patient’s clinical environment [1, 6]. An experienced emergency clinician and an ambulance practitioner ‘take the Emergency Department to the patient’ in an equipped response car [1, 6]. With the use of advanced diagnostics, therapeutics and community services, the patient is often managed within the community and admitted to hospital only when specific needs cannot be safely or optimally met by other services [1, 6]. The range of clinical presentations is broad, reflecting the wide range of urgent and emergency presentations that would be assessed and managed within a typical Emergency Department (ED) [6].

CEM provides an important paradigm shift in how patients presenting with emergency care needs are considered and managed within the health system. Positioning an experienced hospital emergency clinician earlier in the patient’s episode of care allows a senior decision-maker to consider the patient and their needs within the wider health system before they are referred to hospital [6]. Prehospital management supported by alternative community care pathways reduces emergency department attendances by encouraging definitive care within the community complemented with elective hospital services. This model is believed to strengthen existing relationships between primary and secondary care by encouraging cooperative management and providing an opportunity to develop novel clinical pathways. As a consequence, this develops a greater perspective of emergency healthcare for trainees and other staff involved in the patient’s care [1, 6] thereby benefitting other patients in the future.

In 2020, during the initial stages of the COVID-19 pandemic, oncology patients were considered an at-risk group. The London Physician Response Unit (PRU), one of the more mature CEM services, worked in partnership with the Barts Health Acute Oncology Service to provide high-quality care to this vulnerable group through a novel pathway [7]. Oncology patients who sought help via the Chemotherapy Hotline (CHL) and would otherwise have been advised to attend an ED were referred instead to the PRU. Patients are assessed in their homes by an emergency physician with diagnostics and input from their oncologist. Patients requiring hospitalisation are transferred directly to the oncology ward with relevant treatment commenced in the community. However, many of these patients do not require admission. Their care is continued in the community with any further investigations coordinated in an elective manner through the Cancer Acute Assessment Unit (CAAU) [7].

This is an example of a novel pathway that was developed by a CEM service responding to both the needs of the patient and the health system. This type of emergency patient would not be triaged to a PHEM service as their acuity would not reach the threshold. However, their complexity would have resulted in a transfer to hospital through traditional ambulance-based pre-hospital care were it not for a CEM service [7].

### Quality measurement in healthcare

The benefits of CEM have been demonstrated on a local basis with observational data: reduced ED attendances; increased ongoing community care; reduced ambulance utilisation; and an overall cost saving in patient care [6].

However, there has been criticism of the validity of these proposed benefits, based on the data from which they were derived [8]. Such criticism should be respected and reflected upon. This consideration is particularly pertinent for novel health services that deliver care in ways that deviate from traditional practice.

New models of care should facilitate the production of data which can be assessed in a robust and transparent manner. Quality measurement is now widely recognised as an integral component of healthcare service improvement [9–11] and should be used to inform key stakeholders such as clinicians, executives and commissioning bodies. This enables services to develop and mature in purposeful directions. The value of the quality measurement is recognised within pre-hospital care and has been identified within the literature as an area of high research priority [10].

Quality measurement relies on the use of Quality Indicators (QI), concise elements of a service that can be objectively measured [11]. The performance of emergency services has historically been measured by crude, non-clinical, surrogate markers of success such as response times, time to assessment and length of stay. These became popular as they are easily measured and can be understood by a variety of stakeholders including the layperson [12]. However, pre-hospital and emergency care is increasingly complex and so such indicators, when considered alone, offer limited insight into the quality of the care that is delivered [9]. In recent decades there has been significant progress within prehospital care to develop more comprehensive, evidence-based quality indicators that are thought to align more closely with the quality and performance of modern healthcare models [13–17]. Many frameworks and guides now exist to help define and map this more comprehensive approach to quality measurement [10, 14, 18].

Donabedian [18] considers three broad domains of a healthcare service within which quality can be measured: structure, processes and outcomes. Structure refers to the infrastructure within which the healthcare is delivered; processes describe the ways in which care is delivered; and outcomes reflect any change in the patients’ status. This simple conceptual approach remains widely used within initiatives that approach modern healthcare improvement [13, 16, 19, 20].

A wide array of definitions for ‘quality of care’ exist. Establishing exactly what defines ‘quality of care’ is acknowledged to be extremely difficult [21]. One of the more widely cited definitions is from the Institute of Medicine (IOM)-‘Quality of care is the degree to which health services for individuals and populations increase the likelihood of desired health outcomes and are consistent with current professional knowledge [22]. To help contextualise ‘quality of care’, and in recognition of the challenges in defining it, some organisations consider quality by attributes reflective of quality within healthcare systems [9, 21]. The IOM considered a set of six discrete dimensions that could be measured: timeliness, safety, efficiency, equity, effectiveness and patient-centredness [10].

- *Safe—*avoiding harm to patients from the care that is intended to help them. A new model of care must not cause harm with respect to traditional systems.
- *Effective—*providing services based on evidence to all who could benefit and refraining from providing services to those not likely to benefit. The long-term viability of CEM relates to the understanding of where its benefit lies.
- *Patient-centred—*providing care that is respectful of and responsive to individual patient preferences, needs, and values and ensuring that patient values guide all clinical decisions.
- *Timely*—reducing harmful delays for both those who receive and those who give care.
- *Efficient*—avoiding the unnecessary waste of equipment, medication and ideas. CEM must also work efficiently within the health system itself and avoid optimise the use of partner healthcare services.
- *Equitable*—providing care that does not vary in quality because of personal characteristics such as gender, ethnicity, geographic location, and socio-economic status.

We can combine the concepts derived from Donabedian and the IOM and create a ‘quality classification framework’ that can be used to help conceptualise, define and map the use of QI within healthcare (Table 1) [8, 10]. If we were to consider a simple example of ‘time to assessment by clinician’, this would be measuring a *process* within the system, and it would be a measure of *timeliness* (Table 1) [8, 10].

**Table 1:** Quality measures

	Institute of medicine dimensions						
Donabedian `domains		Timeliness	Safety	Efficiency	Equity	Effectiveness	Patient Centredness
	Structure						
	Process						
	Outcome						

This framework is not felt to fully reflect total ‘quality of care’ with respect to CEM. However, it is an acceptable base from which to build. Some argue that stakeholders must consider a quality improvement framework and its domains with respect to their own organisational aims and objectives [21]. This will be considered with the results of this scoping review.

### Aims

It is imperative that we consider how quality measurement may be applied to CEM.

The purpose of a scoping review as opposed to a systematic review of the literature is to summarise a broader overview of existing knowledge, map this knowledge to various concepts and identify any potential gaps of knowledge in this area.

This scoping review aims to identify and present a broad overview of how quality could be measured within a service that delivers CEM.

This scoping review will describe the study characteristics from which the QIs have been developed but an evaluation of the evidence of each QI is beyond the scope of this review.

### Objectives

It aims to develop an understanding of how the results may be applied to CEM, identify gaps in the literature and guide future steps in research. To this end, the review will

1. Map QIs to their respective ‘quality classifications’
2. Consider the populations and study type from which QIs have been derived
3. Identify key themes of QI measurement and disease specific QI
4. Consider themes of QIs that fall outside of the described framework and how they might help develop a quality framework more specific to CEM

## Methods

The methodology follows the guidance provided in the Preferred Reporting Items for Systematic Review and Meta-Analysis extension for Scoping Reviews (PRISMA-ScR) [13]. (See Appendix 3 for PRISMA-ScR checklist). It is also guided by the work of Arksey and O’Malley [23] and the subsequent guidelines developed by the Joanna Briggs Institute [18].

This scoping review protocol has been registered with the Open Science Framework platform (osf.io/e7qxg).

### Protocol stages

1. Develop a research question.
2. Define eligibility criteria.
3. Describe the search strategy and define information sources.
4. Describe the method for data extraction.
5. Explain how the results will be synthesised and reported.

### Research question and framework

To help define the research question and search strategy, we have considered our question using the PCC (participants, concept, context) framework.

### Participants

The participants are defined as the patient populations served by CEM. Patients are adults and children with emergency care needs. The definition of what constitutes an ‘emergency’ situation is difficult to define. The patient’s symptoms (both physical and psychological) may conflict with the perspective of the clinician. However, CEM services should respect the patients’ concerns and expectations where appropriate. In accordance with care in the community, this review will use a holistic approach when considering what constitutes an ‘emergency’. It is acknowledged that the population of CEM is not consistently defined in the literature.

### Concept

Described or defined measures of quality or performance within services that deliver emergency care. The measures/indicators do not need to be in active use and may simply be conceptual. For example, it may have been hypothesised that measuring the rate of hospital admissions in the future for a CEM service is worth considering.

### Context

The context is best defined as physician-based prehospital care.

CEM is delivered in the ‘prehospital’ phase of management; this may also be described as ‘community care’. Historically it was described as the ‘prehospital’ phase of their management with emergency medical services (EMS). However, considering that a significant proportion of these patients are not transferred to the hospital for definitive care it may be better reflected with the term ‘community’. Care could be regarded as both ‘community’ and ‘pre-hospital’, the difference between these at times is nuanced and often dependent on historical categorisation. Care is delivered by senior emergency physician(s) (registrar grade or higher) or other clinician(s) with training similar to that of an emergency physician. An example would be an advanced nurse practitioner under the direction/supervision of a physician lead. The use of such clinicians is a key component of CEM.

### Research question

How can quality be measured within a Physician-Led Community Emergency Medicine Service?

### Eligibility criteria

#### Inclusion criteria

All studies that consider quality measures for the following:

- CEM or pre-hospital care or services that deliver similar care
- Published literature from 1966 onwards [3]
- Clinical and/or non-clinical quality measures
- Adults and children

#### Exclusion criteria

- Trauma. This patient cohort is primarily managed by other services such as PHEM or the ambulance service.
- Diseases with an established pre-hospital protocol. ST-segment Elevation Myocardial Infarction and Cerebrovascular Accidents are managed within pathways and systems that favour immediate disposition to a specialist centre for definitive management.

## Search strategy and information sources

Four sets of search terms based on the PCC question framework have been developed. Context will be considered as two search terms, the setting, i.e. ‘pre-hospital’ and the staff, i.e. physician-led care. This process has involved CEM representatives with terms subsequently reviewed with a librarian.

Search terms will be modified to include standardised vocabulary within each individual database. Additional files describing this process in more detail and a draft search of MEDLINE are provided (Appendix 1).

The search strategy will involve three stages.

1. Initial search: Undertaken using MEDLINE and EMcare. The pilot search will allow a subsequent revision of the search strategy to ensure all of the relevant literature has been captured and that the most appropriate headings have been used within the data extraction tables (Tables 1, 2, and 3). We will analyse the first 10 articles that meet the inclusion criteria including the referenced literature within them. This will allow for the introduction of new appropriate search terms not originally considered [23–25]. Any revision of the search strategy at this point will be documented and explained.
2. Second search: After this pilot is performed and the search strategy reviewed, a full search of MEDLINE will be carried out followed by searches of the online electronic databases: Embase, EMCare, CINAHL, Scopus and the Cochrane Library. A search of other grey literature will be completed. Websites from relevant large organisations such as the National Ambulance Service and London Air Ambulance will be reviewed. Discussions with specialists who represent specific patient groups to hand-pick potentially relevant literature. These specialists have collaborated with the London PRU. A search of Google Scholar will be conducted.
3. Final search: The reference list of all included articles will be reviewed for articles that meet inclusion but are not captured by our second search.

**Table 2:** Data extraction table

Author	Year	Citation	Study design	Population	Proposed Quality Measure	Quality measure Classification	Clinical Sub-type grouping

**Table 3:** Clinical sub-types

Clinical sub-type	Quality measure description	Quality measure classification
Critical care + anaesthesia		
Elderly care		
Oncology		
Palliative care		
Surgical care		
Urology		
ENT		
Infectious disease		
Musculoskeletal		
Paediatrics		
General emergency medicine		

### Data screening process and extraction

Search results will be exported first into Endnote^TM^ and then transferred into Covidence 1(Covidence systematic review software) where all publications will be screened to ensure they fulfil the eligibility criteria, first based on the title, second based on the abstract. This will be performed by two primary reviewers, independently (JS and PMcC).

The remaining articles will be read independently by the primary two reviewers, (JS and PMcC). In the case of uncertainty, the text will be re-evaluated by a third independent reviewer. Data will be stored and charted using Covidence.

A flow diagram in keeping with PRISMA [26] (Appendix 2) will be used to report the searches and inclusion/exclusion pathway.^.^

A data extraction table will be created and used to chart information from each publication (Table 1). Refinement of this table will be allowed for, following the results of the pilot search.

Information of interest will include the following:

1. Study characteristics: year of publication, study type, setting, population
2. Quality measurement classification: Donabedian and IOM classification.
3. Clinical subtype if applicable: mapping to clinical case types attended
4. Themes or grouped categories of measurement

Each of the included studies will be extracted by the two primary reviewers independently and any conflicts resolved through discussion. If there is any ongoing dispute, this will be resolved by the opinion of a third independent reviewer. Authors of publications will be contacted in the event that information is unclear.

### Data synthesis and reporting of results

Results will be summarised descriptively and in tabular form.

Each quality measure will be listed as short descriptive sentences followed by its quality classification and, if clinical, the clinical subtype it represents (see Table 1).

The quality measurement classification will be described in words and coded for reference.

- First by the Donabedian area:
  1. a—Structure, 1. b—Process,1.c—outcome
- Secondly the IOM dimension of quality:
  2. a—Safety, 2. b—Effective, 2. c—Patient-centred, 2.d. Timeliness, 2.e. Efficient, 2.f—Equity

As an example, an indicator that measures ‘desaturation during intubation’ describes an outcome relating to safety and would be classified as 1.c 2.a.

An accurate classification of a QI depends on the details describing the measurement and the context of its application or setting. If we take a proposed measure of ‘effective triage’; one example may be, ‘*The number of cases that are appropriately triaged to a specified team*’. This is an outcome which primarily measures the efficiency of resources. If a similar measure read, ‘*proportion of triage calls in which a representative is actively interrogating calls for OHCA’*, this relates to ‘structure’ and is a measure of efficiency, effectiveness and safety. In instances where more than one dimension is measured, a ‘primary’ IOM dimension is chosen for use in the framework. A balance must be struck when classifying ‘conceptual’ QIs which may lack both detail and context. Each QI will be classified appropriately, to allow comparative analysis.

The total quantity of each specific ‘QI classification’ from the articles extracted (Table 1), will be tallied into the quality classification framework (tabulated framework matrix of Donabedian domains and IOM dimensions). This is a visual illustration, highlighting the breadth of measurements across each classification.

‘Themes’ of quality measures are of interest. Each indicator was grouped into a summarised ‘theme of quality measure’. For example, if a group of QIs assess various time-based measures they were grouped together as ‘job cycle times’.

Clinical data available from local CEM services was reviewed; clinical subtypes based on what is attended by these services were defined and grouped. These groups defined the clinical sub-types: 1. Critical Care and Anaesthesia; 2. Elderly Care; 3. Oncology; 4. Palliative Care; 5. Surgical Care; 6. Urology; 7.ENT; 8. Infectious Diseases; 9. Musculoskeletal; 10. Paediatrics; 11. General Emergency Care/Other.

This data extraction aims to describe what is being measured within current ‘prehospital medicine’ and how it may be applied to CEM. While CEM is a distinct entity within prehospital care, it hopes to draw from literature from other models of care that manage emergency patients within pre-hospital or community settings. To provide an informed base knowledge of the literature, the following will be described and displayed: the source of articles, the populations on which the QIs have been based, the themes of measurement, the breadth of QI classification and gaps that exist within the literature (Table 3).

Understanding these concepts will help contextualise the results and provide direction for the next stage of the research.

## Discussion

This scoping review is the first phase of a larger piece of research designed to develop a set of accredited national QI for use within CEM services. While CEM shares similarities with Emergency Medicine and Prehospital Emergency Medicine it is distinct from both in its aims and delivery. Embedding quality measurement specific to its aims with a quality improvement structure is just one of many fundamental building blocks that will help CEM strategically mature into an established model of emergency healthcare.

The purpose of this scoping review is to provide a comprehensive review of quality measures that could be used within CEM. The results will be mapped using a framework and identify gaps in the literature to help guide future-focused research.

It will act as a base from which to build on recognition that CEM. It will hopefully help us develop our own framework, most relevant to CEM.

Consideration will be given to the integrity and reliability of the data. A set of accredited QIs, utilised nationally within CEM services, is the ultimate aim of this research. A consensus study involving CEM services within the UK and Ireland will form the next phase of this research. The results of this scoping review aim to provide a relevant and broad base of quality measurement to facilitate this study.

### Supplementary Information

**Additional file 1:** **Appendix 1.** Search strategy.

**Additional file 2:** **Appendix 2.** PRISMA flow diagram.

**Additional file 3:** **Appendix 3.** Preferred Reporting Items for Systematic reviews and Meta-Analyses extension for Scoping Reviews (PRISMA-ScR) Checklist.

## Data Availability

As this is a protocol, there are no data available.

## Abbreviations

CEM: Community Emergency Medicine
PHEM: Pre-hospital Emergency Medicine
UK: United Kingdom
NHS: National Health Service
CPR: Cardiopulmonary resuscitation
QI: Quality indicators
IOM: Institute of Medicine

## Footnotes

1 Covidence—an internet-based software program that facilitates independent collaboration among reviewers during the study selection process. This software will also identify and allow removal of duplicates and produce a schematic diagram of each of the selection stages.
