# Supplementary material for: How can quality be measured within a physician-led Community Emergency Medical service? A scoping review protocol
Source: Syst Rev. 2024 Jan 2;13:3. doi: 10.1186/s13643-023-02424-w (PMC10759743; doi:10.1186/s13643-023-02424-w)
Supplement: Supplementary file 1 — Additional file 1: Appendix 1. Search strategy. [file 13643_2023_2424_MOESM1_ESM.docx]

## Search Strategy

1. Participants

Emergency Physician/ or Physician/ or Doctor/ or Advanced care practitioner / Advanced Nurse Practitioner/ Advanced Practice Practitioner / or ACP/ or ANP / or APP/ or Medical Specialist/ or (Physician* or Doctor*or Advanced practitioner*) TI, AB, KW. (*Standard Vocabulary)*

1. Concept

Quality Indicators/ or Quality Measures/ or Performance Indicators / Or Performance Measures/ Or KPIs / or QI / or Effectiveness Measures/ or benchmark Indicators/ or (benchmark* or Quality Indicators* or performance indicators*). TI, AB, KW. (*Standard Vocabulary)*

1. Staff/Persons

Emergency Physician/ or Physician/ or Doctor/ or Advanced care practitioner / Advanced Nurse Practitioner/ Advanced Practice Practitioner / or ACP/ or ANP / or APP/ or Medical Specialist/ or (Physician* or Doctor*or Advanced practitioner*) TI, AB, KW. (*Standard Vocabulary)*

1. Setting

Prehospital /or Pre-hospital/ or Ambulance/ or car/ or (helicopter* or ambulance* or car or vehicle) TI, AB, KW, or (Out-of-hospital or prehospital* or community or Prehospital* emergency medicine or Community Emergency medicine). TI, AB, KW, or (*Standard Vocabulary)*

Each database followed the following search plan.

1. (concept 1)

2. (concept 2)

3. (concept 3)

4. (concept 4)

5. (1 AND 2 AND 3 AND 4)
