# Supplementary figures and images for: How can quality be measured within a physician-led Community Emergency Medical service? A scoping review protocol
Source: Syst Rev. 2024 Jan 2;13:3. doi: 10.1186/s13643-023-02424-w (PMC10759743; doi:10.1186/s13643-023-02424-w)

**Appendix 2 – PRISMA flow diagram**


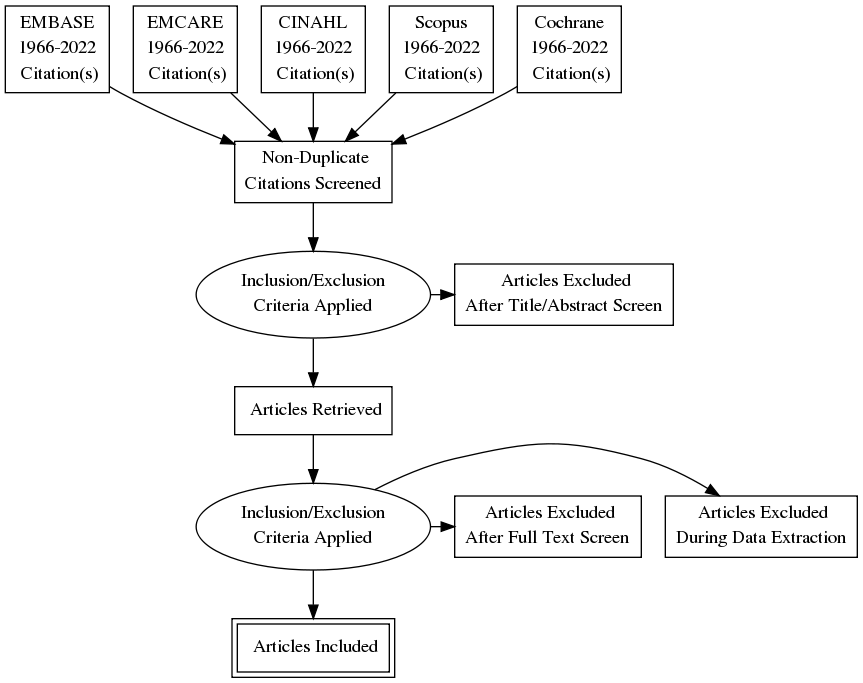

Supplement: Supplementary file 2 — Additional file 2: Appendix 2. PRISMA flow diagram. [file 13643_2023_2424_MOESM2_ESM.docx]
